# Miscellaneous Items

**Published:** 1886-02

**Authors:** 


### MISCELLANEOUS ITEMS.

**Priests** acted as Physicians in Ancient Phœnecia.

**F. M. M.**—No, not of the American stamp, or kind.

**S. R.**—We have not been able to find it yet. Perhaps we may.

**Hermes** was according to Mythology, the founder of medicine in Egypt.

**J. G. M.**—Not before April. They cannot be had at this season of the year.

**The** Israelites embalmed 1,700 years before Christ and probably many centuries earlier. The Egyptians much earlier.

**J. S. Y.**—You must state plainly and briefly exactly what you want. We cannot spend any time in looking up uncertainties.

**It** is stated that castor oil applied daily for two or three weeks, will cure warts. Try it, it will not cost much in time or money.

**Herodotus** said. "Every place is full of doctors in Egypt,' and if he had lived in these days he might have said the same thing of America.

**A Chicago** naturalist says that rats are often troubled with the tricana spiralis, and that hogs often eat them as food and that the tricana is again developed in the hogs from this cause.

**Recent** investigation show that alcohol is oxidized in the system, and is converted into carbon dioxide and water, while but little is eliminated as alcohol from the system.

**Celery** is one of the latest remedies for rheumatism. It is to have stalks and roots boiled together and drink the water freely. It is said that it is vouched for by many who have tried it.

**The** foundation of three-fourths of all cases of consumption is laid before the age of twenty-five years in women mostly in their teens, and by indiscretions in regard to damp, cold impure air. Less than one-fourth of all cases of consumption are due to hereditary causes.

**Will Small-Pox Protect from Vaccinia?** This question has been answered in the negative by the experience of a number of practitioners during the recent numerous vaccinations. Five patients who had well-marked pits of variola on their faces, have been vaccinated, on whom the virus operated, producing undoubted vaccinia. It is settled, then, that even if vaccinia will protect against or modify small-pox, the converse is not true.

**Dr. Austin Flint, Sr.**, at his clinic said digitalis is not indicated either in palpitation or hypertrophy of the heart. The other members of the Bellevue Hospital College, however say it is indicated. Dr. Flint has made great mistakes in his time. We remember one at Buffalo some years ago.

**Vicks Floral Guide.**—We have had ours stolen. It was too good to keep ; but will say what we should have said, if we could have kept long enough to read it, that it is a perfect gem, and recalls to our mind the time when the first of the Vicks commenced his business in Rochester. What a change from that time. The Vicks have grown with time, and now produce this richly filled magazine for all who cultivate flowers, you cannot well get along without it. If you have no room for cultivating them you should have the guide and annual so as to appproximate as near to the cultivation as possible. For they bring you almost into the actual presence of flowers. Send for it. Rochester, N. Y.

**Treatment of Frozen Persons.**—Medical men have always differed as to whether the best medical treatment of frozen persons was by a gradual or rapid application of heat. "To settle the matter," says Knowledge, "Laptchinskki has made a series of very careful experiments upon dogs, with the following results: Of 20 animals treated by the method of gradual resuscitation in a cold room, 14 perished; of 20 placed at once in a warm apartment, 8 died, while of 20 immediately put into a hot bath, all recovered." The experiments will probably influence the practice of medical men in Russia and northern Europe, where the question of the best means of restoring life in persons suffering from excessive cold is of frequent occurrence every Winter.—*Medical Summary.*

**Caulocorea.**—I have found this a very valuable remedy in the debilities common to the female, as a nervine, tonic and alterative. The following will illustrate its remedial applications: Mrs. L., aged 46, who has been losing flesh for years past, had suffered loss of appetite, sleeplessness and nervous debility, with indigestion, and at times great laxativeness of the bowels, and had tried various remedies with little or no success. At last she was recommended the use of Caulocorea, with good results. Now she has better flesh, improved appetite, and more cheerful mind, and is so much pleased with this remedy that she desires to continue its use. Many other cases might be cited to sustain the reputation of this valuable remedy, if it were necessary, as it is frequently and favorably alluded to by medical writers.—J. J. CALDWELL, M. D.

